# Congenital adrenal hyperplasia in the Nordic countries – a potential base for long-term outcome studies

**DOI:** 10.1016/j.lanepe.2023.100616

**Published:** 2023-03-14

**Authors:** Anna Nordenström, Henrik Falhammar

**Affiliations:** a Department of Women's and Children's Health, Karolinska Institute, Stockholm, Sweden; b Paediatric Endocrinology Unit, Karolinska University Hospital, Stockholm, Sweden; c Department of Molecular Medicine and Surgery, Karolinska Institute, Stockholm, Sweden; d Department of Endocrinology, Karolinska University Hospital, Stockholm, Sweden

For rare disorders a national cohort comprising all patients opens-up valuable possibilities to study the long-term effects of the disease. Thanks to the national personal identification numbers the Nordic countries have a unique possibility of linkage between different population-based registers. The national personal identification numbers were introduced many decades ago followed by the creation of different national registers has made evaluations of trends over the years possible.^1^ An example on such a unique study is the article in *The Lancet Regional Health – Europe* by Berglund et al.^2^ The authors have identified all patients diagnosed with congenital adrenal hyperplasia (CAH) in Denmark since 1977 and then confirmed the diagnose by reviewing the medical records.

CAH is a rare group of disorders affecting one or several adrenal enzymes in the steroid producing pathways resulting in impaired cortisol production.^3^ The most common variant of CAH, 21-hydroxylase deficiency (21OHD), also leads to varying degrees of aldosterone deficiency and increased production of adrenal androgens depending on the severity of the disease. Thus, females with classic 21OHD are born with virilised external genitalia while males with classic 21OHD have normal genitals. If the neonate with the most severe form of CAH, i.e., salt-wasting (SW) phenotype, is not identified and treated with glucocorticoids within the first few weeks of life a potentially lethal salt-wasting crisis will occur.^3^ A few percent of residual enzyme activity will result in the slightly less severe the simple virilising (SV) phenotype with considerably less risk of salt losing crisis. Individuals with the SV form are typically diagnosed in the neonatal period in girls due to the virilisation of external genitalia and in the boys later during childhood due to precocious puberty. The mild non-classic (NC) variant has no prenatal virilisation and mostly normal cortisol and aldosterone production but development of androgen excess over time.^4^ In most high-income countries nowadays neonatal screening has been introduced to facilitate diagnosing the neonates with classic CAH, i.e., SW and SV forms.^3^

In the study by Berglund et al. the maximal average prevalence in Denmark was 1/7000 in females and 1/11,000 in males which were in the higher range of international prevalence studies based on data from neonatal screening programs.^2^^,^^3^ The Danish prevalence based on clinical diagnosis was higher than that in Sweden where 1/11,200 were found to have CAH in the neonatal screening.^5^ It should be noted that 92% of the cases detected in the Swedish study were classic CAH. The neonatal screening programs are not designed to pick up all cases with NCCAH, thus most are found in late adolescence or young adulthood, and many are probably never diagnosed, especially among men.^6^ The increased prevalence found by Berglund et al. can be explained by individuals with NCCAH found after the neonatal period but also delayed diagnosed females and males with SV.

It is interesting to note that the median age for diagnosis in the study by Berglund et al.^2^ was in females 3.1 (IQR 1.2–6.6) years and in males 4.8 (IQR 3.2–6.9) years with no apparent change after the introduction of neonatal screening 2009. However, the authors are using the phenotype to classify the severity of CAH which may be challenging. According to the old definition patients with symptoms of CAH after 60 months were classified as NCCAH.^6^ It is not uncommon to develop clitoromegaly over time in females with NCCAH.^7^

Genotyping makes for a more objective and robust way of classifying the degree of severity. A future study correlating the genotype and phenotype as defined here is likely to reveal that a number of individuals may belong to a different genotype group. This may also change the interpretation of the data. For example, it has repeatedly been shown that late diagnosed children, also without growth acceleration, may belong to the I172N genotype group, or they may develop salt crises during an infection. The variation in degree of genital virilization among females with the I172N genotype group is large, ranging from Prader 3-4 to virtually no prenatal virilization. Patients in the I2G genotype group most often have the salt-wasting form but nevertheless present with a slightly different phenotype than patients in the null genotype group. It is also difficult to clinically distinguish between the SV and NCCAH phenotype especially for individuals with the P30L mutation as the mildest allele.

Another contributing factor to the late diagnosis of SVCAH in the Danish population study could be poor performance of their neonatal screening program to detect SVCAH, also pointed out by the authors. In fact, no patient with SVCAH was diagnosed in the Danish neonatal screening during 2009–2018 while 6 cases of SV born during that period were diagnosed later.^8^ In comparison, in Sweden only 2 cases with SV were missed in the neonatal screening during 9 years while at least 22 cases with SVCAH were detected.^5^

The Danish group has established a nice large cohort of patients with CAH with the potential to perform large population-based cohort studies using national registries and matched controls similar to the ones done in Sweden.^9^, ^10^, ^11^ This is a great opportunity to further investigate long-term outcomes in CAH and due to the large number of patients with CAH included also hard endpoints which are normally difficult to study in CAH. We note that the *CYP21A2* genotype was known for 80% of the patients in the Danish study.^2^ Classification using this *CYP21A2* genotype could potentially facilitate comparisons and collaborations across both Denmark and Sweden since both countries have similar personal identification numbers and national diagnosis registries. This would further strengthen the power for comparisons between the different severity groups. Moreover, since these studies will include all/almost all patient with CAH in the countries potential selection bias will be minimised.

## Contributors

Both authors contributed equally.

## Declaration of interests

H Falhammar reports grants from Magus Bervall Foundation and Lisa and Johan Grönberg Foundation as well as consulting fees from Neurocrine Biosciences Inc, Diurnal Limited, Roche Diagnostics International Ltd and Adrenas Therapeutics.
